# Assessment of the efficacy of tuina on treating cervicogenic headache: A protocol for systematic review and meta-analysis

**DOI:** 10.1097/MD.0000000000026224

**Published:** 2021-06-04

**Authors:** Xin Qian, Dongyang Ma, Jiayi Liu, Ting Pan, Haili Wang, Zhe Hu, Zhaohui Wang, Wei Qi

**Affiliations:** a Department of Acupuncture and Tuina, Changchun University of Chinese Medicine, Changchun; b Bao’an Authentic traditional chinese medicine Therapy Hospital, Shenzhen, China.

**Keywords:** cervicogenic headache, protocol, systematic review, tuina

## Abstract

**Background:**

Cervicogenic headache is a secondary headache characterized by unilateral headache, symptoms, and signs of neck involvement. It is often worsened by neck movement, sustained awkward head position, or external pressure over the upper cervical or occipital region on the symptomatic side. In this systematic review, we aimed to evaluate the efficacy and safety of massage therapy for the treatment of cervicogenic headache.

**Methods:**

We searched the China National Knowledge Infrastructure, Chinese Scientific Journal Database, Wanfang Database, China Doctoral Dissertations Full-Text Database, China Master's Theses Full-Text Database, Cochrane Central Register of Controlled Trials, PubMed, and Embase. We will select all eligible studies published on or before April 1, 2021. We will use Review Manager 5.4, provided by the Cochrane Collaborative Network for statistical analysis. We then assessed the quality and risk of the included studies and observed the outcome measures.

**Results:**

This meta-analysis further confirmed the benefits of tuina in the treatment of cervicogenic headache.

**Conclusion:**

The purpose of this meta-analysis was to explore the effect of tuina on patients with cervicogenic headache and to provide more options for clinicians and patients to treat cervicogenic headache.

**Ethics and dissemination:**

This systematic review will evaluate the efficacy and safety of tuina in the treatment of cervicogenic headache. Since all the data included were published, the systematic review did not require ethical approval.

**Registration number:**

INPLASY202150053.

## 1 Introduction

Cervicogenic headache (CEH) is a secondary headache characterized by unilateral headache, symptoms, and signs of neck involvement.^[1,2]^ Sjaastad first put forward the concept of “cervicogenic headache” at the World Headache Congress held in 1983. In 1990, the International Headache Committee formally put forward the diagnostic criteria for cervicogenic headache and made the latest revision in 2009. CEH is a headache caused by disorders of the cervical vertebra and its components such as bone, intervertebral disc, and/or soft tissue, often but not always accompanied by neck pain. Because of its complex and diverse symptoms and lack of specific examination methods, it is often misdiagnosed as neurovascular headache, neurovascular headache, caused by head neurological dysfunction, and vasoconstriction disorder. Most patients are often accompanied by palpitations, insomnia, sweating, and other symptoms, but are also easily misdiagnosed as cardiac neurosis and psychogenic headache. In recent years, the incidence of cervical spondylosis has been increasing year by year and has a younger trend, and people have paid increasing attention to CEH. Therefore, CEH treatment is particularly important.

At present, oral non-steroidal anti-inflammatory drugs, local injection of analgesics, percutaneous radiofrequency neurotomy, nerve block therapy, and high cervical arthrodesis are mainly used in the treatment of CEH, and the curative effect is relatively rapid. However, the side effects of surgery and drug treatment are inevitable, and the recurrence rate is high. In addition, these treatments are expensive and difficult for the general public to bear for a long time; therefore, it is necessary to find cheaper and safer treatments. According to research, massage can significantly improve the symptoms of patients with CEH, produce a sense of comfort, and reduce the occurrence of major accidents and adverse events.^[3,4]^

As a kind of medical means, massage belongs to supplementary and replacement therapy and is widely used.^[5]^ First, massage helps to promote blood circulation and strengthen the physique to achieve the purpose of prevent and treat diseases. Second, existing research shows that the comfort produced by local massage, through peripheral nerve conduction to the central nervous system, will make people have a sense of serenity and relaxation. Finally, massage therapy has the characteristics of simple operation, no obvious adverse reactions, and low price.^[6]^ Many studies have shown that massage can effectively relieve the symptoms related to CEH.^[7–11]^

However, to the best of our knowledge, there is no systematic review of the effectiveness of massage therapy in the treatment of CEH. Therefore, the purpose of this study was to evaluate the efficacy of massage in the treatment of CEH.

## 2 Methods and analysis

### 2.1 Design and registration of the review

This systematic review protocol has been registered on INPLASY. The registration number is INPLASY202150053. The protocol was structured in accordance with the guidelines of the Preferred Reporting Items for Systematic Reviews and Meta-analyses Protocols (PRISMA-P).^[12]^

### 2.2 Inclusion criteria for study selection

#### 2.2.1 Types of studies

Only randomized controlled trials (RCTs) were published or registered before April 1, 2021. Quasi-randomized controlled trials, review articles, case reports, and other studies that do not meet the requirements will be excluded.

#### 2.2.2 Types of patients

The patient's age was between 18 and 70 years. In view of the fact that there is no unified standard for the diagnosis of this disease, the diagnostic criteria adopted in this study were based on the diagnostic criteria of cervicogenic headache issued by Sjaastad on behalf of the International Cervical Headache Research Group in 1998, and the latest revision of the diagnostic criteria for cervicogenic headache by the International Headache Committee in 2009, and combined with clinical formulation, headache is accompanied by ipsilateral cervicoccipital pain, with or without shoulder and arm non-root pain. The initial onset was mostly unilateral intermittent or persistent pain. The tenderness of the upper cervical segment was obvious and the neck muscles were tense; the compression of the occipital area and high cervical vertebra area of the affected side could induce headache; the activity of the neck was limited and the activity could induce headache; the nerve block test was positive; the patient exclusion criteria included the presence of other diseases resulting in serious cognitive or speech disorders; patients who could not understand and complete the therapist's instructions (Mini-Mental State Examination <21 points), history of drug or alcohol dependence, serious liver or kidney disease, other diseases that may affect brain structure and function, and other mental disorders.

#### 2.2.3 Interventions types

The experimental group received tuina treatment, while the control group adopted treatments generally approved for treating CEH, such as oral medication, physical therapy, behavioral therapy, or acupuncture.

### 2.3 Outcome measures

#### 2.3.1 Primary outcome

The primary outcome measure will be the visual analogue scale pain score.

#### 2.3.2 Secondary outcomes

Secondary outcomes will include range of motion (ROM) score and headache duration.

#### 2.3.3 Article exclusion criteria

Studies with the following conditions were excluded: repeated data or data that could not be extracted, observational studies, retrospective studies, non-random trials, quasi-experimental studies, and animal studies. In addition, studies with insufficient data or a lack of effective classification were not included.

### 2.4 Search methods for the identifying of studies

English and Chinese search strategies will be conducted on 8 databases: the China National Knowledge Infrastructure, Chinese Scientific Journal Database, Wanfang Database, China Doctoral Dissertations Full-Text Database, China Master's Theses Full-Text Database, Cochrane Central Register of Controlled Trials, PubMed, and Embase. In addition, we will conduct manual retrieval of conference papers, ongoing experiments, and internal reports, among others, to supplement electronic retrieval. We will select all eligible studies published on or before April 1, 2021.

### 2.5 Search strategy

The search strategy will be based on the Cochrane handbook guidelines (V.5.1.0) including keywords such as “cervicogenic headache,” “tuina,” or “massage,” and “RCT.” Subsequent searches will use Medical Subject Headings (MeSH) headings, including “cervicogenic headache,” “tuina,” and “massage,” in addition to keywords from the initial retrieval. Additional article searches will review the reference lists of relevant research articles. As an example, the search strategy for PubMed is summarized in Table 1.

**Table 1:** Search strategy for PubMed.

No	Search terms
#1	massage. ti,mesh.
#2	massage therapy. ti,ab.
#3	tuina. ti,ab.
#4	or #1-#3
#5	cervicogenic headache. ti,ab.
#6	Headache. ti,ab
#7	or #4-#5
#8	randomized controlled trial. pt.
#9	controlled clinical trial. pt.
#10	randomized. ab.
#11	randomly. ab.
#12	trial. ab.
#13	or #8-#12
#14	exp animals/not humans. sh.
#15	#13 not #14
#16	#4 and #7 and #15

### 2.6 Data extraction

#### 2.6.1 Study selection

**Figure 1:**
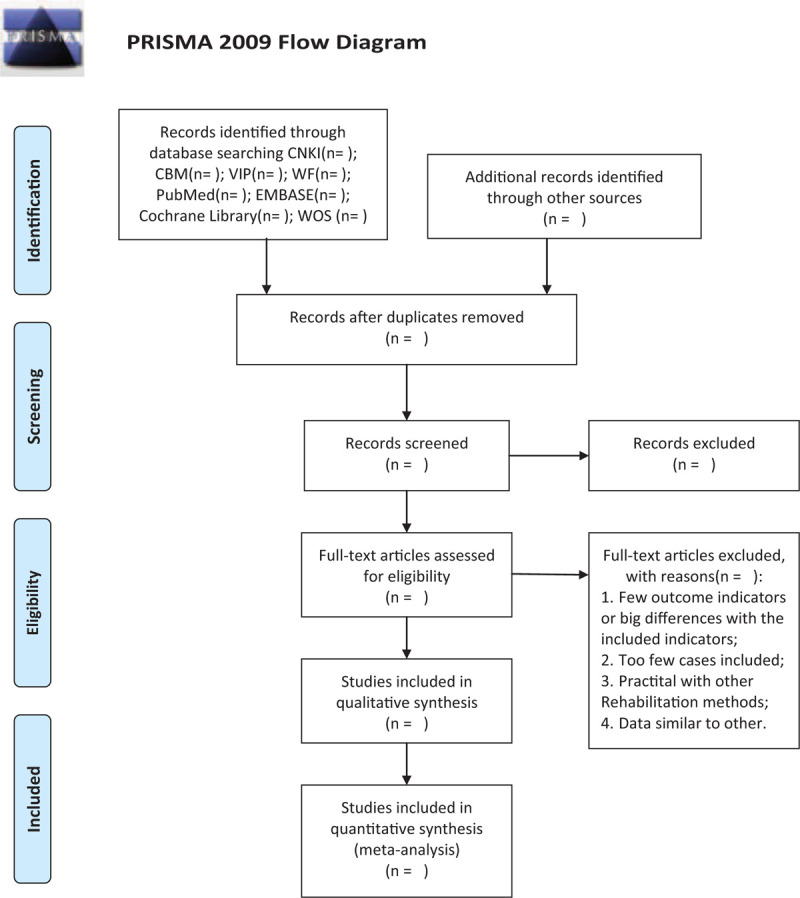
PRISMA flow diagram of study and exclusion. PRISMA = Preferred Reporting Items for Systematic Reviews and Meta analyses.

The records from the database and other resources will be uploaded to the database created in EndNote V.9.7. All excerpted abstracts were independently screened by the review authors (XQ and DM). We will obtain the full text of all articles that may be suitable for further evaluation of eligibility based on inclusion/exclusion criteria. Studies that did not meet the inclusion criteria were excluded, and the reasons for exclusion were recorded. Any differences were resolved through consensus or discussion with a third party (WQ). The final selection process followed the PRISMA guidelines,^[13]^ as shown in Fig. 1.

#### 2.6.2 Data extraction and management

Two reviewers (XQ and DM) will independently apply the inclusion and exclusion criteria to assess the eligibility of each retrieved study. The following data were extracted from the selected studies using the data collection table and recorded in an Excel file: first author and publication year, study design, sample, intervention measures, type of measures, bias risk assessment, and research results. The results will be cross-checked by the 2 reviewers, the differences will be resolved by consensus, and any ongoing differences of opinion will be arbitrated by the third reviewer (JL). If necessary, we can contact the original author to provide more relevant information. The data extraction form included the following items:

1. General information: title, author, year of publication and research area, average patient age, average course of disease, and treatment time.
2. Trial characteristics: design, follow-up time, randomisation method, allocation concealment, incomplete result data, and blindness (patients, people receiving treatment, result evaluators).
3. Intervention: primary intervention (routine rehabilitation treatment, application time, and session duration); comparison interventions (routine rehabilitation treatment, application time, and session duration).
4. Patients: total number of people in each group, total number of people, baseline characteristics, diagnostic criteria, prognosis, and loss to follow-up (reasons, descriptions).
5. Results: Main results, adverse reactions, adverse reaction time, follow-up time, and quality of results report.

#### 2.6.3 Risk of bias in assessment

Two reviewers (TP and HW) will independently apply the bias tool from the Cochrane Handbook for Systematic Reviews of Interventions to evaluate the risk of bias in each selected study.^[14]^ Six dimensions will be evaluated: random sequence generation, allocation hiding, blinding of patients, researchers, and outcome evaluators, incomplete outcome data, selective reporting, and other issues. These studies will be divided into 3 quality levels: low risk of deviation, high risk of deviation, and risk of unclear deviation.^[12]^ Any discrepancies were resolved through discussion with a third author. When a consensus cannot be reached through discussion, a third-party reviewer (WQ) will make a decision.

#### 2.6.4 Treatment effect measures

The method varies depending on the type of data. For dichotomous variables, total effective rates, and adverse events, we will analyze the rate ratio. For continuous variables, we will analyze mean differences. The 95% CI was presented for both dichotomous and continuous outcomes.

#### 2.6.5 Missing data management

We will contact the original author to obtain missing or incomplete data and wait for a reply within 1 month after sending the email. If missing data cannot be obtained, incomplete data will be excluded from the analysis.

#### 2.6.6 Heterogeneity assessment

Statistical heterogeneity was assessed using *I*^2^ statistics.^[15]^ An *I*^2^ statistic <50% indicates that the level of statistical heterogeneity is low; ≥50% will be considered as significant statistical heterogeneity. If substantial heterogeneity is found, we will report it and use sensitivity analysis and subgroup analysis to explore possible causes.

#### 2.6.7 Reporting biases assessment

If the included studies exceeded 10 trials, we constructed a funnel chart to assess reporting bias. Otherwise, the Egger test will be performed using Stata V.15.1 software (Stata Corp, College Station, TX, USA).

#### 2.6.8 Subgroup analysis

If possible, we plan to carry out the following group analysis: study regional differences, differences in conventional rehabilitation methods, differences in average course of disease, and differences in treatment time. We will use Review Manager V.5.4 (The Cochrane Collaboration, Oxford, England), in the formal test of subgroup interaction.

#### 2.6.9 Sensitivity analysis

When possible, we will conduct a sensitivity analysis to explore the impact of the trial's risk of bias on the preliminary results. These analyses will exclude lower-quality trials and repeat meta-analyses based on sample size and insufficient data to assess quality and robustness when significant statistical heterogeneity occurs.

### 2.7 Grading the quality of evidence

The online version of the Grading of Recommendations Assessment, Development, and Evaluation methodology (GRADE; https://www.gradeworkinggroup.org/) will be used to assess the quality of the evidence and risk of bias, categorized into 4 levels: high, moderate, low, or very low.^[16]^

### 2.8 Ethics and dissemination

This review will evaluate the efficacy and safety of massage for the treatment of CEH. Since all included data will be obtained from published articles, ethical approval is not required and will be published in peer-reviewed journals. Due to the lack of systematic review in this field, this study will be combined with relevant randomized controlled trials to better explore the evidence of massage in the treatment of CEH and guide clinical practice and massage research.

### 2.9 Patient and public involvement

This article is based on previous research and does not involve any patient and public participation, nor does it involve any new research conducted by the author on human subjects.

## 3 Discussion

With the aging of the world population and the impact of changes in living habits and environment, cervicogenic headache has become a common health problem in daily life, and it is a disease to be solved in the clinic, because its diagnosis is often unclear and prone to repeated attacks. At present, oral non-steroidal anti-inflammatory drugs and local injection of analgesics are mainly adopted, but the side effects of these methods are inevitable; the recurrence rate is high, and the cost is high.

Tuina promotes blood circulation and strengthens the physique to achieve the purpose of prevent and treat diseases. In addition, existing research shows that the comfort produced by local massage, through peripheral nerve conduction to the central nervous system, will make people have a sense of serenity and relaxation. In addition, tuina therapy has the characteristics of simple operation, no obvious adverse reactions, and low price. Many studies have shown that massage can effectively relieve the symptoms related to cervicogenic headache.

However, the specific mechanism for the treatment of cervicogenic headache requires further research. This systematic review and meta-analysis will provide patients, clinicians, and medical policy makers with a better understanding of the efficacy and safety of tuina in the treatment of CEH. We hope to provide credible evidence and valuable medical references for tuina treatment of CEH.

## Author contributions

**Conceptualization:** Xin Qian, Zhaohui Wang.

**Data curation:** Xin Qian, Dongyang Ma.

**Formal analysis:** Jiayi Liu, Ting Pan, Haili Wang.

**Funding acquisition:** Wei Qi.

**Investigation:** Ting Pan, Haili Wang.

**Methodology:** Xin Qian, Zhaohui Wang.

**Supervision:** Wei Qi.

**Validation:** Xin Qian, Dongyang Ma, Zhe Hu.

**Writing – original draft:** Xin Qian.

**Writing – review & editing:** Xin Qian, Zhaohui Wang, Wei Qi.
